# Case Report: Anti-glomerular basement membrane disease during pregnancy with favorable renal outcome, sequential biopsies, and dual anti-α1/α3(IV) and anti-LM521 antibodies

**DOI:** 10.3389/fimmu.2026.1714790

**Published:** 2026-06-01

**Authors:** Jing Zhuang, Siwen Gu, Yue Qu, Changrong Zhang, Huang Kuang, Zhao Cui, Xuefei Tian, Hong Jiang

**Affiliations:** 1 Division of Nephrology, Department of Internal Medicine, People’s Hospital of Xinjiang Uygur Autonomous Region, Urumqi, China; 2 Renal Division, Peking University First Hospital, Beijing, China; 3 Institute of Nephrology, Peking University, Beijing, China; 4 Key Laboratory of Renal Disease, Ministry of Health of China, Beijing, China; 5 Key Laboratory of Chronic Kidney Disease (CKD) Prevention and Treatment, Ministry of Education of China, Beijing, China; 6 Section of Nephrology, Department of Internal Medicine, Yale University School of Medicine, New Haven, CT, United States

**Keywords:** anti-GBM disease, anti-laminin-521 antibody, anti-type IV collagen α1/α3 antibody, kidney biopsy, pregnancy

## Abstract

Anti-glomerular basement membrane (anti-GBM) disease during pregnancy is rare and often life-threatening. We report a G4P1 woman who developed atypical anti-GBM disease at 13 weeks of gestation. Serial kidney biopsies demonstrated a reduction in active necrotizing lesions from 64.3% to 30.8% following early intensive immunosuppression and pregnancy termination. Western blot identified circulating autoantibodies against type IV collagen α1/α3 chains and laminin-521 (LM-521)—a novel finding in anti-GBM disease during pregnancy. Multidisciplinary management, including plasma exchange, high-dose methylprednisolone, hemodialysis, pregnancy termination, and obinutuzumab for serologic recurrence, enabled discontinuation of dialysis and partial renal recovery with stable kidney function. This case provides histological evidence, through sequential kidney biopsies, that early intensive immunotherapy combined with pregnancy termination can halt active necrotizing lesions and facilitate tissue repair. It also examines the potential association between specific autoantibody subtypes (anti-α1/α3 chains and LM521) and disease phenotypes, offering new insights into anti-GBM disease during pregnancy.

## 1 Introduction

Anti-glomerular basement membrane (anti-GBM) disease during pregnancy remains a rare but clinically challenging condition with significant knowledge gaps. First, most reported cases rely on single-timepoint kidney biopsies (1, 2), which fails to capture the dynamic histopathological evolution of the disease during treatment. Second, the detection of anti-GBM antibodies is usually based on conventional enzyme-linked immunosorbent assay (ELISA), which carries a documented false-negative rate of more than 7% (3), potentially delaying timely diagnosis and intervention. Third, there is no consensus regarding optimal therapeutic strategies, as treatment decisions must balance maternal survival and renal recovery with fetal well-being.

We describe a unique case of anti-GBM disease presenting at 13 weeks of gestation. Sequential kidney biopsies allowed direct observation of the pathological evolution from acute necrotizing glomerulonephritis to a convalescent phase characterized by reduced active necrotizing lesions. This case demonstrates the potential for histological repair in pregnancy-associated anti-GBM disease and highlights that early, intensive immunosuppressive therapy combined with timely obstetric intervention can substantially mitigate disease activity. Collectively, these findings provide histological evidence supporting timely, individualized therapeutic approaches in this high-risk setting.

## 2 Case report

### 2.1 Clinical presentation

A 33-year-old Chinese unemployed woman, gravida 4 para 1 (G4P1), presented on March 29, 2024, at 13 weeks of gestation with cough, sputum production, low-grade fever (37.8 °C), headache, and dizziness, without identifiable precipitating factors. Urinalysis revealed proteinuria and microscopic hematuria. After ineffective empirical anti-infective therapy, she was referred to our institution. Her medical history was notable only for hyperthyroidism. She had no prior kidney disease, psychiatric illness, or relevant family history. On admission, she appeared anemic with coarse breath sounds bilaterally. Laboratory testing showed moderate anemia, markedly elevated inflammatory markers, and acute kidney injury. Chest imaging demonstrated bilateral pulmonary infiltrates. Fetal ultrasound confirmed a single intrauterine live fetus with growth appropriate for gestational age.

### 2.2 Diagnosis and treatment

Immunological evaluation revealed a weakly positive anti-GBM antibody titer of 1:10 (institutional reference range: < 1:10). Concurrent testing for anti-neutrophil cytoplasmic antibodies (ANCA), anti-Sm, anti-dsDNA, and other autoantibodies was negative, and the antistreptolysin O level was normal. These findings clinically and serologically excluded ANCA-associated and immune complex-mediated crescentic glomerulonephritis.

**Figure 1:**
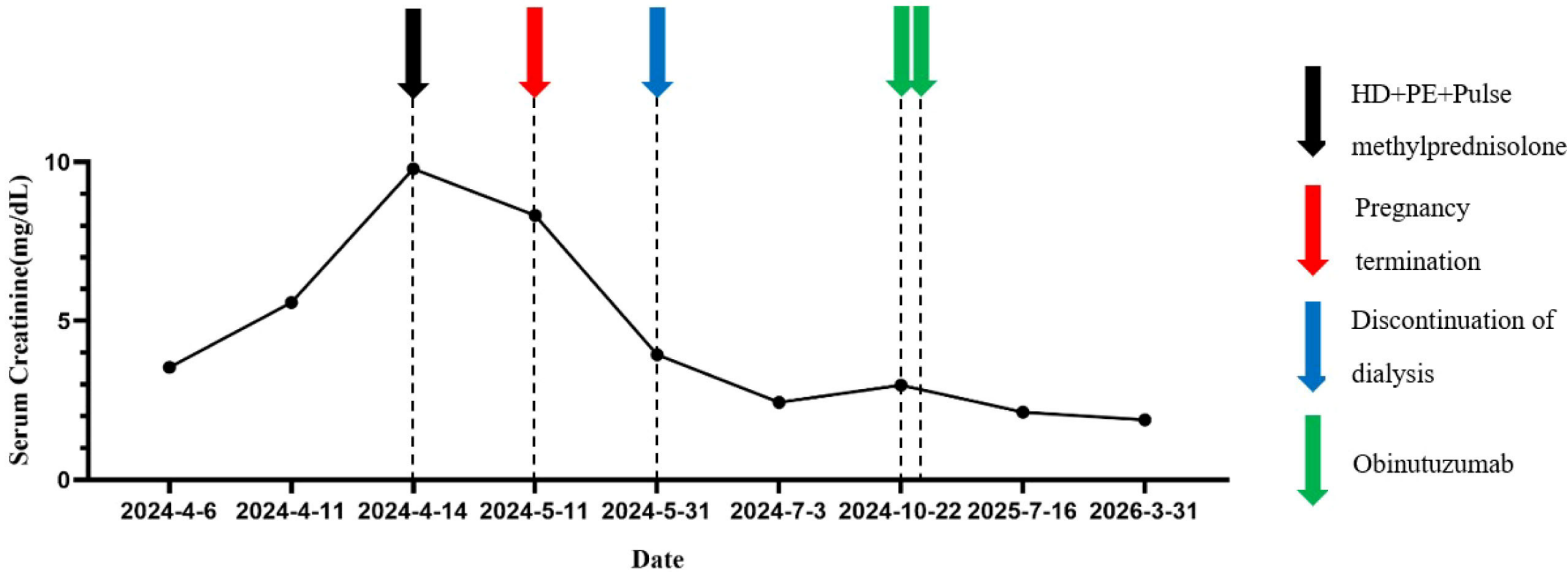
Changes in serum creatinine levels during the observation period. HD, hemodialysis; PE, plasma exchange.

**Figure 2:**
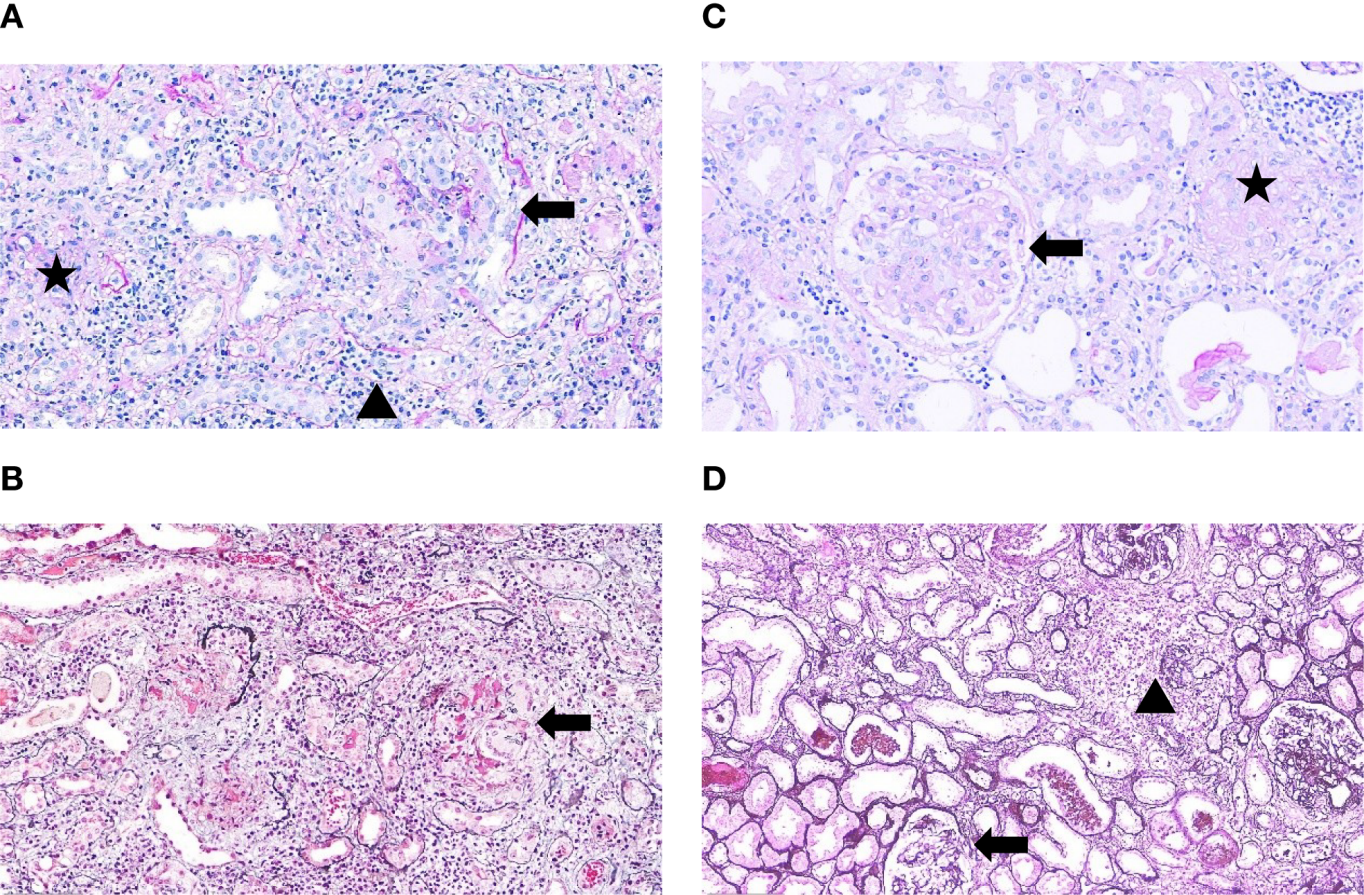
Dynamic histopathological evolution across sequential kidney biopsies. **(A)** Acute phase biopsy: A representative glomerulus exhibiting severe active lesions, including extensive rupture of Bowman’s capsule (↑), segmental fibrinoid necrosis (★), and diffuse interstitial inflammatory infiltrates (▲). Periodic acid–Schiff (PAS), ×200. **(B)** Acute phase biopsy: Another glomerulus demonstrating widespread rupture of Bowman’s capsule (↑), a hallmark of catastrophic structural destruction in active anti-GBM disease. Periodic acid–methenamine silver (PAM), ×200. **(C)** Convalescent phase biopsy (3 months post-onset): A representative glomerulus showing preserved Bowman’s capsule integrity (↑) and the formation of a fibrous crescent (★), reflecting the transition from active necrosis to tissue organization and histologic repair. Note the significantly attenuated interstitial inflammation. PAS, ×200. **(D)** Convalescent phase biopsy: Demonstration of arrested active necrotizing lesions, characterized by segmental glomerulosclerosis (↑) and mild chronic interstitial fibrosis (▲), indicating the stabilization of structural damage. PAM, ×200.

Despite supportive care with antibiotics and albumin infusion, the patient remained oliguric without significant clinical improvement, and her serum creatinine rose progressively to 5.58 mg/dL (**Table 1**; **Figure 1**). A kidney biopsy performed on April 11, 2024, with informed consent, yielded 14 glomeruli. Histopathological examination demonstrated necrotizing glomerulonephritis with Bowman’s capsule rupture in 64.3% (9/14) of the glomeruli, accompanied by acute interstitial nephritis-establishing a definitive diagnosis of active anti-GBM disease despite the weak initial serological signal (**Figures 2A, B**).

**Table 1:** Temporal evolution of key clinical indicators.

Parameter	Respiratory presentation phase (2024-04-06)	First kidney biopsy (2024-04-11)	Dialysis phase (2024-04-14 to 2024-05-31)	Second kidney biopsy (2024-07-03)	Follow- up period (2024-10-22)	Post-obinutuzumab (2025-07-16)
**Blood pressure** (mmHg)	127/72	111/65	121/73	129/76	113/73	100/69
Kidney function						
Urine output (mL/day)	450	550	800	1400	2400	2200
Urinary RBC (/HPF)	50-60	40-60	50-60	40-60	35-45	N/A
Proteinuria (g/24h)	1.09	N/A	1.65	0.70	0.79	0.38
Serum creatinine (mg/dL)	3.54	5.58	9.79	2.44	2.98	2.13
Pulmonary status						
PaO_2_ (mmHg)	71.6	N/A	N/A	N/A	94	91.4
Inflammatory markers						
Leucocyte (10^9^/L)	11.25	11.12	11.29	7.53	8.34	6.38
Neutrophils(%)	79.8	81.9	79.7	58.6	48.1	58.5
C-reactive protein (mg/L)	148.19	125.59	94.53	8.09	1.17	0.73
Procalcitonin (ng/mL)	8.88	5.99	4.02	0.04	0.08	0.05
**Serum albumin (g/L)**	24.7	28.7	29.7	34.6	42.2	40.2
**Anti-GBM antibodies**	Weak^+^	Weak^+^	Negative	Negative	1:10 titer^++^	Negative

^+^Weak positive by ELISA. ^++^Positive by ELISA with reported titer. N/A, not assessed; RBC, red blood cells; HPF, high-power field.

The bold values: focused examination.

As kidney function further deteriorated with serum creatinine peaking at 9.79 mg/dL, the patient was treated with intravenous methylprednisolone pulses (0.5 g/day for 3 days), daily plasmapheresis, and hemodialysis. Following the pulse therapy, she was maintained on an intravenous infusion of methylprednisolone at 40 mg/day. At 18 weeks of gestation, a multidisciplinary team weighed the maternal renal prognosis against fetal viability. After extensive counseling and informed consent, the pregnancy was terminated. Subsequently, her kidney function improved sufficiently to allow the discontinuation of dialysis. Intermittent follow-up testing for ANCA remained consistently negative throughout the disease course.

A second kidney biopsy, obtained three months after the initial presentation during the recovery phase, sampled 26 glomeruli. Histological evaluation revealed a significant attenuation of active necrotizing lesions compared to the initial biopsy (**Table 2**).

**Table 2:** Summary of histopathological features across the sequential kidney biopsies.

Pathological features	First biopsy	Second biopsy
Total glomeruli (n)	14	26
Glomeruli with active fibrinoid necrosis (n, %)	9 (64.3%)	8 (30.8%)
Crescents (n, %) *	1 (7.1%)	5 (19.2%)
Globally or segmentally sclerotic glomeruli (n, %)	2 (14.3%)	6 (23.1%)
Interstitial fibrosis	0	<25%
Tubular atrophy	<25%	<25%

*Includes cellular, fibrocellular, and fibrous crescents. Interstitial fibrosis and tubular atrophy reflect the percentage of cortical involvement, based on semi-quantitative evaluation by a renal pathologist.

At the three-month follow-up, the patient experienced a serologic recurrence, characterized by the reappearance of anti-GBM antibodies. Concurrently, her serum creatinine level increased from 2.44 mg/dL to 2.98 mg/dL. Further serological evaluation, performed according to the protocol described by Kuang et al, identified circulating autoantibodies targeting the type IV collagen α1/α3 chains and laminin-521 (LM-521)—the first such finding reported in anti-GBM disease during pregnancy. Additionally, an in-house ELISA was utilized to screen for antibodies against all five type IV collagen α chains (detailed methodology is provided in the **Supplementary Material**) (4, 5). Given the catastrophic nature of the patient’s initial presentation and the high risk of irreversible nephron loss associated with anti-GBM disease, we aimed to proactively prevent a full clinical relapse. Consequently, she was treated with obinutuzumab (1000 mg × 2 doses, administered two weeks apart) combined with low-dose oral prednisone (10 mg/day). This regimen successfully achieved antibody seroconversion and stabilized her kidney function (**Table 1**; **Figure 1**). At the most recent follow-up on March 31, 2026, her serum creatinine had further decreased to 1.89 mg/dL.

The patient did not experience any significant discomfort during the diagnosis, treatment, and follow-up process. She felt that her overall physical condition had improved significantly and had no complaints at all. She remains under regular follow-up.

## 3 Discussion

Anti-GBM disease during pregnancy is a rare but catastrophic clinical emergency, characterized by complex pathophysiology and a generally dismal maternal-fetal prognosis. Historically, patients presenting with severe acute kidney injury requiring dialysis face an extremely high risk of irreversible end-stage kidney disease (ESKD) and fetal loss. Despite this, our patient’s serum creatinine progressively decreased, her anti-GBM antibodies turned negative, and her urine output was preserved (1000–1500 mL/d), ultimately allowing her to become dialysis-independent.

A comprehensive literature review of the PubMed, Web of Science, EMBASE, and Cochrane databases (through April 2026) identified only 18 reported cases of anti-GBM disease during pregnancy (detailed in the **Supplementary Material**). To our knowledge, this is the first report documenting dynamic histopathological changes through sequential kidney biopsies in this setting.

At presentation, establishing an accurate diagnosis was challenging. The initial kidney biopsy findings, the absence of pulmonary hemorrhage, and the weakly positive circulating anti-GBM antibodies were consistent with atypical anti-GBM nephritis. During the diagnostic workup, we clinically and serologically ruled out ANCA-associated glomerulonephritis and immune complex-mediated crescentic glomerulonephritis. Following an individualized regimen of plasmapheresis, hemodialysis, corticosteroids, and pregnancy termination, the patient’s kidney function improved sufficiently to discontinue dialysis. The second biopsy demonstrated a significant reduction in active crescents (down to 30.8%), indicating a transition toward histologic repair. Although active necrotizing inflammation was successfully arrested, the second biopsy also revealed an inevitable progression in chronicity, marked by an increase in globally sclerotic glomeruli and mild interstitial fibrotic changes. This histopathological transition from acute necrosis to fibrotic tissue repair perfectly mirrors her clinical course: successful discontinuation of hemodialysis, yet stabilization at a stage of chronic kidney disease (CKD) with serum creatinine maintained at a higher baseline (2.13–2.98 mg/dL), rather than complete functional normalization. However, this favorable outcome cannot be attributed solely to early intensive immunosuppression. The rapid clinical improvement strongly suggests that terminating the pregnancy played a vital physiological role. Pregnancy termination likely relieved the massive renal hemodynamic burden typical of gestation. Furthermore, high estrogen levels during pregnancy may drive Th2 responses and autoantibody production (6), while the delivery of the placenta leads to a sharp decline in pro-inflammatory mediators such as IL-6 and TNF-α (7, 8). Together, these interventions created a critical window that halted active necrosis and facilitated tissue healing.

Another notable finding was the patient’s complex serological profile. Initial ELISA testing yielded only a weakly positive result, a method known to carry a false-negative rate of approximately 7% (9). Indeed, some patients with atypical anti-GBM nephritis remain seronegative across ELISA, indirect immunofluorescence, and immunoblotting (10). n our patient, Western blot analysis conducted five months post-onset identified autoantibodies against type IV collagen a1/a3 chains and LM-521' to 'In our patient, ELISA conducted five months post-onset identified autoantibodies against type IV collagen a1/a3 chains and LM-521—antibody subtypes not typically seen in classical anti-GBM disease. The α1(IV) chain is more abundant in alveolar basement membranes, and anti-α1(IV) antibodies are usually linked to pulmonary hemorrhage in anti-GBM disease and lung adenocarcinoma (11). LM-521, composed of α5, β2, and γ1 chains, is essential for maintaining the glomerular filtration barrier (12). Kuang et al. previously described an anti-GBM nephritis patient harboring anti-LM-521 antibodies without anti-α3(IV), while animal studies confirmed that LM-521 immunization induces crescentic nephritis (5). The severe, kidney-limited injury in our patient, despite the absence of pulmonary hemorrhage, implies that antibody heterogeneity strongly influences organ-specific phenotypes. However, because these novel antibodies were identified during the follow-up period rather than in the acute phase, we must exercise interpretive restraint. We cannot definitively conclude they were the primary initiating trigger. It is plausible that their emergence represents intermolecular epitope spreading—a phenomenon where the severe acute architectural destruction of the GBM exposes previously sequestered cryptic antigens. This novel serologic observation expands the known autoantibody spectrum of anti-GBM disease, but further research is necessary to clarify their precise pathogenic role.

During follow-up, the patient experienced a serologic recurrence. To minimize the risk of irreversible damage to her remaining viable nephrons, and considering the life-threatening nature of the disease, we adopted a proactive strategy. Although some case reports describe successful deliveries following rituximab therapy without sustained neonatal B-cell depletionion (13), our team utilized obinutuzumab to control the recurrence. Obinutuzumab is a type II anti-CD20 monoclonal antibody that induces deeper tissue-resident B cell depletion, and it effectively achieved seroconversion without adverse events. Beyond B-cell targeted therapies, future precision strategies such as epitope modification hold clinical promise. For example, Shi et al. engineered the nephritogenic T-cell epitope of α3(IV)NC1 to generate a peptide that delayed and attenuated kidney injury in experimental anti-GBM glomerulonephritis (14).

## 4 Conclusions

This case provides the first histological evidence of dynamic kidney repair in anti-GBM disease during pregnancy, documented through sequential biopsies. It demonstrates that partial recovery of active necrotizing lesions can be achieved through a synergistic approach involving early intensive immunotherapy and timely pregnancy termination. Additionally, the identification of novel autoantibodies against type IV collagen α1/α3 chains and LM-521 expands our understanding of antibody heterogeneity and potential epitope spreading in anti-GBM disease. Timely histopathological diagnosis, proactive monitoring of serologic recurrences, and individualized management—including the use of novel biological agents like obinutuzumab—can substantially improve clinical outcomes in this rare and high-risk condition.

## 5 Patient perspective

The patient described the diagnosis and rapid deterioration of her kidney function during pregnancy as frightening. She expressed gratitude to the multidisciplinary team for clearly explaining the risks and available options. Although pregnancy termination was the most difficult decision, she believed it contributed to her recovery. She hopes that sharing her experience will help other pregnant women and their physicians make informed decisions earlier.

## Data Availability

The original contributions presented in the study are included in the article/**Supplementary Material**. Further inquiries can be directed to the corresponding author.
