# Supplementary material for: Case Report: Anti-glomerular basement membrane disease during pregnancy with favorable renal outcome, sequential biopsies, and dual anti-α1/α3(IV) and anti-LM521 antibodies
Source: Front Immunol. 2026 Jun 1;17:1714790. doi: 10.3389/fimmu.2026.1714790 (PMC13265547; doi:10.3389/fimmu.2026.1714790)
Supplement: Supplementary file 1 [file SupplementaryFile1.docx]

**Supplementary Material**

**Supplementary Table 1. Summary of clinical and pathological characteristics, treatment responses, and maternal-fetal outcomes in 18 reported cases of anti-GBM disease during pregnancy.**

| **First Author (Year)** | **Age**  **(Years)** | **Gravida/**  **Para** | **Gestational Age at Onset (Weeks)** | **Clinical Presentation** | **Laboratory and Pathological Features** | **Pulmonary Imaging** | **Main Therapeutic Interventions** | **Renal Outcome** | **Pregnancy Outcome** | **Neonatal Outcome** |
| --- | --- | --- | --- | --- | --- | --- | --- | --- | --- | --- |
| Nilssen^1^ (1985) | 19 | G1/P1 | 19 | Hematuria, proteinuria | Anti-GBM (+), crescent formation, linear IgG deposition | Alveolar hemorrhage | PE + MP + CTX + HD | Kidney transplantation | Fetal death (28 wks) | N/A |
| Deubner^2^ (1995) | 21 | G1/P1 | 12 | Hypertension, edema | Anti-GBM, crescentic GN | Not assessed | PE + MP + CTX + HD | Kidney transplantation | Pregnancy termination (35 wks) | N/A |
| Al-Harbi^3^ (2003) | 30 | N/A | 28 | Dyspnea, oliguria | Anti-GBM(-→+), linear IgG (3+) deposition | Pulmonary congestion | HD + MP + PE | Dialysis-dependent | Preterm delivery (34 wks) | SGA  (1.6 kg) |
| Vasiliou^4^ (2005) | 32 | G2/P0 | 18 | Oliguria, frothy urine | Anti-GBM (+), 80% crescents, linear IgG deposition | Alveolar hemorrhage | HD + PE + MP + AZA | Dialysis-independent | Preterm delivery (26 wks) | Brain injury, developmental delay |
| Nair^5^ (2013) | 23 | G1/P0 | 13 | Dyspnea, edema | Anti-GBM >300 mg/dL, 60% crescents | Not assessed | PE + CTX + HD + Pregnancy termination | Dialysis-independent | Pregnancy termination (15 wks) | N/A |
| Adnan^6^ (2016) | 17 | N/A | 6 | Persistent vomiting | Anti-GBM (+), acute necrotizing GN | Not assessed | PE + MP + HD | Dialysis-independent | Spontaneous abortion (8 wks) | N/A |
| Divyaveer^7^ (2017) | 26 | G2/P1 | 12 | Edema, oliguria | p-ANCA (+)/Anti-GBM (+), linear IgG deposition | Normal | PE + MP + HD + CTX | Dialysis-dependent | Medical abortion (12 wks) | N/A |
| Qin^8^ (2018) | 17 | N/A | 13 | Hemoptysis, anuria | Anti-GBM (+), diffuse crescent formation | Pulmonary consolidation | MP + HD | Death | Maternal death (20 wks) | N/A |
| Sprenger-Mähr^9^ (2019) | 30 | G1/P0 | 13 | Hemoptysis, pulmonary embolism | p-ANCA (+)/Anti-GBM (+), 17% active lesions | Not assessed | MP + PE + CTX + RTX | Renal recovery | Term delivery (38 wks) | SGA (2.28 kg) |
| Li^10^  (2021) | 23 | N/A | 18 | Hemoptysis, AKI | Anti-GBM 30.3 AI, no kidney biopsy | Alveolar hemorrhage | PE + HD + CTX + RTX | Dialysis-independent | Preterm delivery (28 wks) | Normal development |
| Lodhi^11^ (2021) | 23 | G3/P1 | 15 | Diarrhea, anuria | Anti-GBM (+), 100% cellular crescents | Not assessed | HD + PE + MP + AZA + TAC | Maintenance PD | Preterm delivery (28 wks) | SGA  (1.1 kg) |
| Kai^12^ (2021) | 28 | G1/P0 | 8 | Edema, oliguria | Anti-GBM/MPO-ANCA (+), 96% fibrocellular crescents | Normal | HD + MP + PE | Dialysis-dependent | Pregnancy termination | N/A |
| Riahi^13^ (2022) | 35 | N/A | 32 | Hemoptysis, dyspnea | Anti-GBM (+), no kidney biopsy | Pulmonary hemorrhage | MP pulses | Renal recovery | Term delivery (39 wks) | Normal |
| Chandra^14^ (2022) | 25 | G1/P0 | 34 | Oliguria, edema | Anti-GBM (+), crescentic GN | Not described | HD + Pregnancy termination + PE + CTX | Dialysis-dependent | Pregnancy termination (34 wks) | N/A |
| Kafagi^15^  (2024) | 20 | G2/P0 | 16 | Hemoptysis, syncope | Anti-GBM 30.3 U/mL, no kidney biopsy | Pulmonary consolidation | PE + MP + HD + RTX | Dialysis-independent | Preterm delivery (28 wks) | Good |
| Jafry^16^ (2024) | 30 | G3/P2 | 27 | Hematuria, vomiting | Anti-GBM 133.3 RU/mL, crescentic GN | Not described | MP + PE + CTX | Antibody decline | Cesarean section (33 wks) | Normal |
| Chandler^17^ (2024) | 20 | G7/P1 | 15 | Severe vomiting | Anti-GBM (-), linear IgG/κ/λ deposition, diffuse crescents | Not assessed | PE + Oral CTX + MP | Dialysis-dependent | Pregnancy termination | N/A |
| Jayanti^18^  (2025) | 36 | N/A | 6 | Dyspnea, fevers, hemoptysis | Anti-PR3 ANCA (+) (titre 150 IU/mL) and positive anti-GBM (titre 58 CU), cellular crescent, segmental fibrinoid necrosis | Diffuse alveolar hemorrhage | PE + MP pulses + CTX + RTX + Pregnancy termination | Renal recovery | Pregnancy termination (8 wks) | N/A |

**Abbreviations:** AKI, acute kidney injury; AZA, azathioprine; CTX, cyclophosphamide; GBM, glomerular basement membrane; GN, glomerulonephritis; HD, hemodialysis; MP, methylprednisolone; PD, peritoneal dialysis; PE, plasma exchange; RTX, rituximab; SGA, small for gestational age; TAC, tacrolimus; wks, weeks; N/A, not available / not applicable.

**2. In-house ELISA Methodology for Type IV Collagen α Chains and Laminin-521 (LM-521)**

As referenced in the main manuscript, an in-house enzyme-linked immunosorbent assay (ELISA) was utilized to detect autoantibodies against all five type IV collagen α chains and LM-521. The specific detection methods were performed as follows:

Briefly, recombinant proteins were coated onto 96-well microtiter plates (Nunc Maxisorp; Thermo Fisher Scientific, Waltham, MA, USA) at a concentration of 2 μg/mL in bicarbonate buffer (pH 9.6) and incubated overnight at 4°C. The plates were subsequently incubated with patient serum samples (diluted 1:100) for 30 minutes at 37°C. Following a rigorous washing step, alkaline phosphatase–conjugated goat anti-human IgG (1:5000 dilution; Sigma-Aldrich, St. Louis, MO, USA) was added and incubated for an additional 30 minutes. Colorimetric development was achieved using a p-nitrophenyl phosphate substrate, and the optical absorbance was read at 405 nm using a microplate reader. The diagnostic cutoff values for positivity were strictly established at the mean optical density plus three standard deviations (SDs) derived from a cohort of healthy controls^19,20^.

**References**

1. Nilssen DE, Talseth T, Brodwall EK. The many faces of Goodpasture's syndrome. *Acta Med Scand*. 1986;220(5):489-91. doi: 10.1111/j.0954-6820.1986.tb02800.x.

2. Deubner H, Wagnild JP, Wener MH, Alpers CE. Glomerulonephritis with anti-glomerular basement membrane antibody during pregnancy: potential role of the placenta in amelioration of disease. *Am J Kidney Dis*. 1995;25(2):330-335. doi:10.1016/0272-6386(95)90016-0

3. Al-Harbi A, Malik GH, Al-Mohaya SA, Akhtar M. Anti-glomerular Basement Membrane Antibody Disease Presenting as Acute Renal Failure During Pregnancy. *Saudi J Kidney Dis Transpl*. 2003 Oct-Dec;14(4):516-21.

4. Vasiliou DM, Maxwell C, Shah P, Sermer M. Goodpasture syndrome in a pregnant woman. *Obstet Gynecol*. 2005;106(5 Pt 2):1196-1199. doi:10.1097/01.AOG.0000161061.35611.98

5. Nair S, George J, Kumar S, Gracious N, Das M. A case of Goodpasture's syndrome complicating pregnancy with dialysis requiring renal failure responding to plasmapheresis and termination of pregnancy. *Ren Fail*. 2013 Sep;35(8):1173-5. doi: 10.3109/0886022X.2013.815566.

6. Adnan MM, Morton J, Hashmi S, Abdul Mujeeb S, Kern W, Cowley BD. Anti-GBM Disease in Pregnancy: Acute Renal Failure Resolved After Plasma Exchange, Hemodialysis, and Steroids. *J Investig Med High Impact Case Rep*. 2016;4(1):2324709615624232. doi:10.1177/2324709615624232

7. Divyaveer S, Tiwari V, Patil M, et al. An unusual case of rapidly progressing glomerulonephritis in pregnancy; “triple positivity” or a co incidence? *J Nephropathol*. 2017;6(4):272-274. doi:10.15171/jnp.2017.44

8. Qin J, Song G, Liu Q. Goodpasture’s syndrome in early pregnancy: A case report. *Exp Ther Med*. 2018;15(1):407-411. doi:10.3892/etm.2017.5425

9. Sprenger-Mähr H, Zitt E, Soleiman A, Lhotta K. Successful pregnancy in a patient with pulmonary renal syndrome double-positive for anti-GBM antibodies and p-ANCA. *Clin Nephrol*. 2019 Feb;91(2):101-106. doi: 10.5414/CN109584.

10. Li AS, Abdullah S, Szeki I, Brix SR. Anti-glomerular basement membrane disease in pregnancy. *J Am Soc Nephrol*. 2021;32:506.

11. Lodhi FAK, Akcan T, Mojarrab JN, Sajjad S, Blonsky R. A Case of De Novo Antiglomerular Basement Membrane Disease Presenting during Pregnancy. *Case Rep Nephrol*. 2021 Mar 17;2021:5539205. doi: 10.1155/2021/5539205.

12. Kai H, Usui J, Tawara T, et al. Anti-glomerular Basement Membrane Glomerulonephritis During the First Trimester of Pregnancy. *Intern Med*. 2021;60(5):765-770. doi:10.2169/internalmedicine.5722-20

13. Riahi T, Najafi L, Asadi Ghadikolaei O, Tajik Jalayeri MH. Goodpasture’s syndrome in pregnancy without renal involvement: A case report. *Caspian J Intern Med*. 2022;13(2). doi:10.22088/cjim.13.2.442

14. Chandra A, et al. A rare case of anti GBM disease presenting in third trimester of pregnancy. *Indian J Nephrol*. 2022.

15. Kafagi AH, Li AS, Jayne D, Brix SR. Anti-GBM disease in pregnancy. *BMJ Case Rep*. 2024;17(4):e257767. doi:10.1136/bcr-2023-257767

16. Jafry NH, Butt N, Mubarak M, Akhtar SF. Anti-glomerular basement membrane disease complicated by malaria during pregnancy with successful maternal and fetal outcomes: a case report. *J Nephrol*. 2024. doi:10.1007/s40620-024-02089-1

17. Chandler S, Palamuthusingam D. Atypical anti-GBM disease in pregnancy. *BMJ Case Rep*. 2024;17(11):e260284. doi:10.1136/bcr-2024-260284

18. Jayanti S, Li J, Renthawa J, Lin MW, Lee V. Double Positive Anti-PR3 ANCA Vasculitis and Anti-GBM Vasculitis in a Pregnant Woman: Case Report. *Nephrology (Carlton)*. 2025;30(9):e70124. doi:10.1111/nep.70124

19. Zhao J, Cui Z, Yang R, Jia XY, Zhang Y, Zhao MH. Anti-glomerular basement membrane autoantibodies against different target antigens are associated with disease severity. *Kidney Int*. 2009 Nov;76(10):1108-15. doi: 10.1038/ki.2009.348.

20. Kuang H, Shen CR, Jia XY, et al. Autoantibodies against laminin-521 are pathogenic in anti-glomerular basement membrane disease. *Kidney Int*. 2023;104(6):1124-1134. doi:10.1016/j.kint.2023.07.023
